# A bibliometric analysis of research on tourism content marketing: Background knowledge and thematic evolution

**DOI:** 10.1016/j.heliyon.2023.e13487

**Published:** 2023-02-04

**Authors:** Phuong Minh Binh Nguyen, Xuan Lan Pham, Giang Nu To Truong

**Affiliations:** a University of Economics Ho Chi Minh City, Ho Chi Minh City, Viet Nam; b School of Management, University of Economics Ho Chi Minh City, Ho Chi Minh City, Viet Nam

**Keywords:** Co-word, Co-citation, Bibliometric, Content marketing, Tourism

## Abstract

Content marketing is becoming an important trend in the tourism industry and is attracting the attention of many stakeholders. Previous studies have sporadically highlighted only some content marketing aspects, but none comprehensively described the topic. To fill this gap, this study adopts a comprehensive approach by combining two bibliometric co-citation and co-keyword analysis methods of 659 articles on content marketing in travel sectors. The co-citation results indicate that tourism content marketing research has been concentrated on the themes of (1) the impact of electronic word of mouth (eWOM) and word of mouth (WOM) on business performance; (2) the role of social media, user-generated content (UGC) and destination image formulation; (3) the impact of eWOM and UGC on the decision-making process; as well as (4) opportunities and challenges. Additionally, based on the co-keyword analysis, hot research topics are explored, including online review implementation; UGC implementation; communication and information search; customer behavior prediction model; the decision-making process; and issues related to user experience, quality, and management. Among these, UGC implementation is the most likely trend that researchers can develop in the future. In addition, the influence of other types of UGC (e.g., user-generated travel videos) is a promising avenue for future research. This study will help researchers understand the role and influence of tourism content marketing. Furthermore, tourism marketers can use content marketing to restore destination image and address last-minute booking issues after the COVID-19 pandemic.

## 1 Introduction

Using content marketing as a tool has become a prominent trend [1]. It generates significant benefits and affects almost every sector and context. The development of digital marketing has made it easier for customers to access product information to support their decision-making process [2], creating favorable conditions for online content to influence customer behavior. In the context of tourism, content marketing (e.g., social media) can influence visitors’ final decisions [3] as it has the potential to portray the destination before tourists visit. To develop tourism and stay current with marketing trends, tourism entrepreneurs or destination businesses must understand the meaning of information-sharing trends and increasingly update their knowledge [4].

In an academic context, considerable research has demonstrated the role of content marketing in the tourism sector. Some focused on innovations and development [ [5,6]]. Although research to date is valuable, practice is constantly changing, thus requiring timely interpretation of the theory. Developing and diversifying research issues related to tourism content marketing is essential. This study provides an overview of the tourism content marketing field as a valuable reference for scholars, especially those new to the field or even tourism professionals interested in and aiming to implement content marketing to develop tourism.

Low and MacMillan [7] p. 139] argued that “as a body of literature develops, it is useful to stop occasionally, take inventory of the work that has been done, and identify new directions and challenges for the future.” However, traditional review methods in tourism were assumed to be dominant [8]. The traditional review is also known as a qualitative or narrative review. In this regard, the application of such review methods was assessed without applicable conditions, and the findings were therefore invalid [9]. A narrative review requires authors to filter through all data records and solely rely on their judgment and perception [10]. Such manual implementation seems subjective and is probably not immune to bias [11]. Meanwhile, a bibliometric literature review method allows the processing of many datasets collected from the research field [12]. The analysis techniques from bibliometrics assist practitioners in processing data entirely using computers, making the process faster, more convenient, and more efficient [13].

Furthermore, the bibliometric method allows the visualization of research data with maps and graphs, which is difficult to achieve with traditional methods [14]. Compared to the traditional literature review method, the bibliometric method may have disadvantages in terms of detailed analysis but an advantage in terms of comprehensiveness. Therefore, these two approaches are complementary rather than antagonistic [13].

Travel content marketing is receiving increasing attention from marketers and scholars [15]. However, for a field to thrive, research must be logically synthesized based on previous literature [16]. Therefore, a quantitative literature review may improve the understanding of a field and reveal, over time, the main discussions in previous studies and their relationships [17]. In addition, Liu et al. [18] concluded that it was crucial to identify existing intellectual structures from scientific publications and focus efforts on topics of great interest in the era as this could improve academic impact and establish scholars’ reputations. Thus, quantitative assessments of research areas that have long-term coverage and reveal vast information are urgently required [18]. Meanwhile, the bibliometric method includes co-citation analysis that allows the identification of knowledge structures based on scientific mapping [19]. Furthermore, a co-keyword analysis can visually outline hot and emerging research trends that scholars can focus on [19].

Based on the above arguments and using the bibliometric method, this study reviewed the existing literature on tourism content marketing. To the best of the authors’ knowledge, this is the first study to deploy a bibliometric review in the research field. The following questions were addressed:

(1) What are the intellectual structures of tourism content marketing studies?
(2) What are the major research themes in tourism content marketing? What are prominent scientific insights most cited to shape each research theme?
(3) What is the most potential theme that could be developed in the future?

The remainder of the paper is organized as follows. The literature review in section 2 covers a conceptual presentation of tourism content marketing and the bibliometric method. The research methodology and an in-depth analysis of the results are presented in sections 3, 4, respectively. Finally, a discussion and concluding remarks, including some limitations and suggestions for improvement, are provided in section 5.

## 2 Literature review

### 2.1 Tourism content marketing

Presently, users are familiar with the concept of content marketing. Content marketing developed due to the explosion of digital marketing, increasingly fierce competition, and the need to draw visitors' attention to information related to products and services [20]. This concept refers to creating and distributing relevant and valuable content to customers to attract and retain them [21]. Content-marketing activities are conducted on online platforms such as corporate websites, forums, virtual communities, blogs, vlogs, social media, and even mobile apps [22]. In tourism, content marketing provides information and knowledge about a tourist destination and shared travel experiences on a particular online platform to potential tourists. Using such content as a reference allows visitors to make effective decisions [23]. On the business side, owing to fierce competition and elasticity of demand, to grow sales, the created content must be appealing and attract visitors' attention [6]. Therefore, tourism businesses’ deployment of a content-marketing strategy that often only mentions the address, location, facilities, and booking information is no longer sufficient. Moreover, the needs and requirements of tourists while searching for information are increasingly demanding and rapidly changing [24]. Thus, having in-depth knowledge and understanding of trends in the field of tourism content marketing is critical. These factors are the basis for businesses to create an effective content marketing strategy that meets the diverse needs of visitors while being unique and exciting.

### 2.2 Bibliometrics

Pritchard [25] suggested that bibliometrics is a statistical and mathematical analysis of bibliographic records. Osareh [26] and Okubo [27] defined it as an analytical tool that includes specific methods for quantifying databases related to scientific publications. Compared to the systematic literature review, this method can avoid author bias [28]. The main advantage is that this method integrates quantitative rigor with subjective literature analysis [19]. The quantitative approach of the bibliometric method allows an author to describe and evaluate scientific knowledge clearly and systematically, improving the assessment quality. In this vein, the bibliometric method is a powerful aid to document reviewers. It directs researchers to the most influential scientific works and helps outline a comprehensive picture of the research map while avoiding subjective bias [19]. Bibliometric methods use various techniques such as co-citation analysis, bibliographic coupling, and co-author and co-keywords analysis [29]. This study adopts only two main analytical techniques: co-citations and co-keyword analyses. The former allows for the determination of both the number of citations of the article by two other papers [19] and the relationship between articles that cite the same document [30]. This technique helps researchers identify the scientific background of topics through relationships created from a co-citation network.

Similarly, co-keyword analysis allows researchers to aggregate the number of occurrences of keywords that appear together in articles [31]. It also creates a network known as a co-keyword map, visualizing the relationships of keywords [32]. Co-keyword analysis techniques effectively identify concepts, trends, and developments in the evaluated research area [32].

Since 2008, bibliometric studies in tourism have grown significantly [33]. Some completed works include those tourism marketing via the Internet [34], social media research in hospitality and tourism [8], trust in hospitality and tourism [35], destination choice [36], and consumers' experience in hospitality and tourism [37]. However, the existing reviews individually reflect aspects of tourism content marketing that fragment the conceptual structure of a subject. Du Plessis [38] concludes that the current research related to content marketing has not fully reflected its components and their impact because scholars approach the topic in different contexts and fields. Furthermore, to the best of our knowledge, no bibliometric study to date has focused on a general analysis of content marketing in tourism using a quantitative approach. Therefore, this study established and outlined a panorama of the field's scientific knowledge and suggests the most likely research trends that scholars can focus on developing. In addition, industry professionals and marketers can use the information to implement effective marketing strategies and policies.

## 3 Methodology

**Fig. 1:**
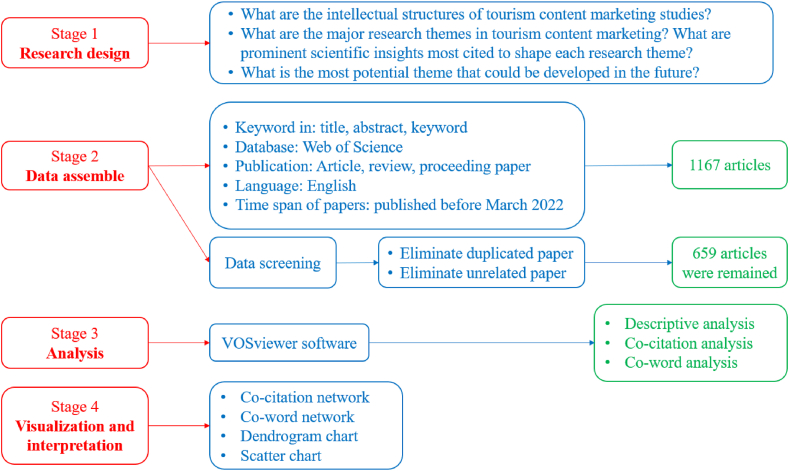
Technical roadmap of the bibliometric method in tourism content marketing.

This study aims to outline the scientific structure and thematic evolution of tourism content marketing. According to Zupic and Cater [19], the primary purpose of the bibliometric method is science mapping, which assists in determining the intellectual structure and thematic development of a scientific field. Zupic and Cater [19] propose an approach that allows processing a large amount of scientific knowledge in a research field and eliminates subjectivity and bias. This approach has been applied in marketing and tourism studies [33] and could be an alternative to qualitative methods or meta-analyses. Therefore, this study utilizes the process proposed by Zupic and Cater [19], including research design, data assembly, analysis, data visualization, and interpretation. The specific process is illustrated in Fig. 1.

### 3.1 Research design

To achieve the research objectives, two main techniques were applied. The first was a co-citation analysis technique that helped answer the first question by determining the intellectual structure of the tourism content marketing field [39] also used co-citation analysis for a similar purpose. Next, the co-keyword analysis technique was utilized, which helped answer the second research question by visually sketching the conceptual structure and the thematic development of the research field, consistent with Muritala et al. [39] and Chiang [40].

### 3.2 Data assembly

English publications related to tourism content marketing (articles, reviews, conference papers) were compiled for review. Data were collected from the Web of Science (WoS) database, which was chosen because it is the most commonly used in bibliometric reviews [19]. In addition, it allows a variety of entries for search conditions with a large sample [41]. This review used the keywords listed in Table 1 to aggregate data. The keywords were defined based on the concepts described in the previous section. The selected publications contained the keywords mentioned in the title/description/keywords sections.

**Table 1:** Keywords searched on Web of Science.

No	Keywords	Number of publications
1	“advertisement” AND “tourism"	135
2	“online content” OR “visual content” AND “tourism"	100
3	“online communication” OR “virtual communication” AND “tourism"	38
4	“content marketing” AND “tourism” OR “tourism content” AND “marketing"	12
5	“generated content” OR “content-based” AND “tourism"	437
6	“eWOM” AND “tourism"	215
7	“online review” OR “online travel reviews” AND “tourism"	230
**Total**		**1167**

Duplicate studies were removed in the data screening stage. Next, the titles, abstracts, and keywords were skimmed to remove articles irrelevant to the research area. Following Zhang et al. [13], the authors independently read all the records in Excel format and noted their retention or removal decisions based on each author's perceptions. Decisions in this screening include:

• Retain: the research was considered entirely relevant to tourism content marketing.
• Consideration: the research was considered potentially relevant to the tourism content marketing sector. Reading the full text was required to make a final decision.
• Remove: the study was completely unrelated to tourism content marketing.
• Not available: although the studies reviewed were “considered,” no single source containing the full text could be found.

Based on the above, the studies categorized as “remove” and “not available” were excluded. Voting was conducted for decisions that had not been reached by consensus from the author group. A retained article was one that most of the authors had accepted.

The authors then conducted quality checks of the publications. In this respect, only peer-reviewed articles published in reputable journals will be retained for further in-depth analysis. The research has compiled scientific data from WoS, so the quality can be guaranteed because selecting and evaluating publications from the input scientific journals is very strict [42]. Therefore, as long as the journals are not on Beall's list, they can be ready for further analysis [43]. The final result was 659 articles relevant to the tourism content marketing field.

### 3.3 Analysis

Data synchronization was conducted prior to data analysis. First, the same author's name, but with a different encoding, was synchronously corrected. For example, “Ye qa” and “Ye q” would be merged into “Ye q.” Additionally, keywords that described the same concept but were encoded differently were also corrected for synchronization. For example, “user generated content” or “user-generated content” or “UGC” would be edited to “user-generated content” (UGC). The data were then analyzed in three stages. The first stage was descriptive analysis. This stage presented the basic information on the 659 retained documents, including the number of publications by year and journals related to the research and geographical area. The next stage was co-citation analysis using VOSviewer software. The results of the analysis generated co-citation clusters. These clusters were labeled to visualize the scientific structure of the research topic. The final stage was co-keyword analysis using VOSviewer software. This analysis divided the studies into chronological periods based on publication date. The thematic groups generated from the co-keyword analysis allowed developments in the research area to be unambiguously described. A scatter plot and hierarchical distribution graph supported using the Power BI tool were outlined to suggest and visually present potential research trends.

## 4 Results

### 4.1 Descriptive analysis

**Fig. 2:**
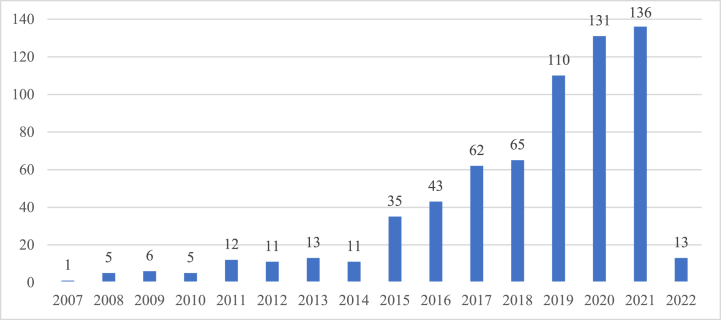
Number of publications by year in the tourism content marketing field.

The time evolution of the scientific publications presented in Fig. 2 indicates that, although the number of studies before 2015 was relatively modest, it grew strongly after 2015. Despite a slowdown in 2017 and 2018, it continued to grow. Since this study was conducted in March 2022, publications related to tourism content marketing have not been fully aggregated. On the basis of the number shown in the chart, it seems that this field of research has still not garnered the sufficient attention/attention it deserves. However, the authors strongly believe that in terms of the number of scientific publications, the field of tourism content marketing is in a period of rapid and robust development, especially at a time when the whole world is stepping up promotional activities and marketing communications to restore tourism following the COVID-19 pandemic. Toubes et al. [44] observed that the tourism industry was the most affected by the COVID-19 pandemic. A large body of research addressed the problems caused by the pandemic. However, its sudden outbreak has opened up many potential approaches, such as alternative marketing solutions (online marketing/content marketing), during this period [44]. Therefore, the number of publications related to the research field may grow strongly.

The next part of the descriptive analysis calculates the number of journal publications. Based on Table 2, *Tourism Management* had the most significant number of publications related to tourism content marketing, with 56 publications. Sustainability followed this with 53 publications and the *International Journal of Contemporary Hospitality Management* with 42 publications.

**Table 2:** Distribution of publications.

Rank	Journals	Number of publications
1	Tourism Management	56
2	Sustainability	53
3	International Journal of Contemporary Hospitality Management	42
4	International Journal of Hospitality Management	35
5	Journal of Travel Research	34
6	Journal of Travel Tourism Marketing	34
7	Current Issues in Tourism	33
8	Journal of Destination Marketing & Management	26
9	Journal of Hospitality and Tourism Technology	23
10	Journal of Vacation Marketing	15
	Others	308

### 4.2 Co-citation analysis

The authors calculated that an average of 43.5 citations out of 659 articles should be retained for the co-citation analysis. Following McCain [45], the authors chose a cut-off point for the analysis. According to van Eck and Waltman [29], co-citation analysis has no standard set of thresholds. However, Ertz et al. [46] concluded that meaningful clusters were best visualized when established at a threshold of 20. Therefore, the selected cut-off point in this review was 20. Hence, with 659 articles retained as input for VOSviewer, the system calculated the citations in their bibliographies to form a co-citation network in which citations appeared together at least 20 times. The results from VOSviewer formed a co-citation network with a total of 118 articles grouped into four clusters, which are believed to have influenced and shaped the research topic.

**Fig. 3:**
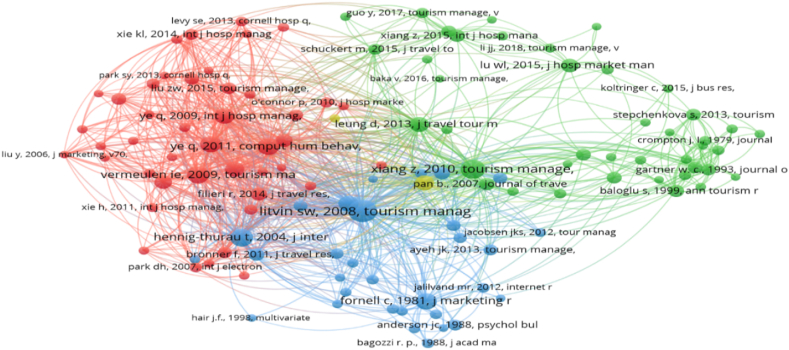
Visualized co-citation network in tourism content marketing.

The co-citation clusters are presented in Fig. 3. Bubbles show the first author's name and publication year: the larger the bubble, the higher the number of citation occurrences. Bubbles of the same color are interpreted as citations belonging to the same cluster. The distance between the two bubbles indicates the relevance and similarity between the two citations. Finally, the thickness of the links indicates the strength of the connection between the citations.

Based on the generated co-citation network, the authors read all the articles in each cluster to identify the main topic of discussion, followed by naming the clusters formed. According to Baker et al. [47], the naming is subjective, depending on the author's perception and objectives but collectively agreeing with the assessment. Based on this rule, the authors named each cluster independently. If the naming results differed among authors, a discussion was held to unify each cluster's name. The naming results are reported in Table 3.

**Table 3:** Co-citation clusters and representative studies as pillars of theoretical foundation.

Cluster name	Prominent citations
C1: The effects of eWOM and WOM on business performance	Vermeulen & Seegers [48]; Ye et al. [49]; Sparks & Browning [50]; Ye et al. [51]; Cantallops & Salvi [52]
C2: The role of social media, UGC, and destination image formation	Xiang & Gretzel [53]; Leung et al. [54]; Lu & Stepchenkova [55]; Baloglu & McCleary [56]; Pan et al. [57]
C3: The effects of eWOM and UGC on the decision-making process	Litvin et al. [58]; Hennig-Thurau et al. [59]; Gretzel & Yoo [60]; Bronner & De Hoog [61]; Ayeh et al. [62]
C4: Opportunities and challenges	Buhalis & Law [63]; O'connor [64]

Cluster 1 was named “The effects of eWOM and WOM on business performance. The similarities among the studies in this cluster include the use of conceptual and theoretical models of eWOM and WOM to account for the effects of customer reviews on hotel booking [48], beliefs and intentions in booking [50], hotel room revenue [51], and restaurant popularity variables [65].

Cluster 2 was named “The role of social media, UGC, and destination image formation.” This cluster includes articles on the role of social media, UGC, destination formation, and data analysis methods of activities related to tourism content marketing. Xiang and Gretzel's article [53] is the most cited study in this cluster. They investigated the role of social media in tourist travel-planning behavior based on visibility in search-engine results. In addition, the role of social media is also considered a destination marketing tool [66] that impacts marketing strategy and tourism management [67].

The role of social media can be approached from both the visitor's and supplier's viewpoints [54]. Some studies in this cluster also adopt big data analysis to focus on the impact of UGC on tourism and hospitality issues [55]. Various types of UGC, such as travel blogs, have also been found to be related to the effectiveness of destination marketing based on semantic network analysis and content-analysis methods [57]. The customer experience (a form of UGC) shared on the Expedia platform is also related to visitor satisfaction through big data and text analytics [68]. Several studies in this cluster discuss forming tourist destination images through content [69] and path analyses [56]. Problems related to destination image formation appear to explain, emphasize, and complement the impacts of tourism content marketing activities, although there are few in the cluster.

Cluster 3 deals with “the effects of eWOM and UGC on the decision-making process.” The studies in this cluster mainly discuss the impact of customers' perceptions and motivations on decision-making to receive or share information or behave toward tourist destinations. The main research subjects in this cluster are UGCs, such as travel reviews, travel commentary, and shared travel experiences. Litvin et al.’s article [58] is the most prominent in this cluster, with the highest number of citations. This study comprehensively outlines the visitor decision-making process, from the visitor's initial motivation from marketing activities emanating from a supplier to perception formation and the tourist's behavior intention. The authors identify several prominent theories mentioned in this cluster as a basis to explain the relationship between motivation, perception, and intention/behavior: equity and balance theory [59], source credibility theory (SCT) model, the theory of homophily [62], social cognitive theory [70], technology acceptance model (TAM), and motivational theory [71]; and the theory of planned behavior (TPB) [72]. Moreover, the TAM and TPB were the two most used theories in research on tourists' UGC acceptance [73]. In this cluster, structural equation modeling is a popular methodology [ [74,75]].

Finally, Cluster 4 is identified as “opportunities and challenges.” The studies in this cluster discuss the opportunities and challenges associated with tourism content marketing, such as innovation, technological development, and tourism-industry functions [63]. In addition, the popularity of UGC creates many benefits for the hospitality industry, but it also raises obstacles such as fake reviews, which businesses need to take seriously to avoid losses [64].

In the four clusters, the authors did not detect any related to the application model of content marketing from the perspective of marketers or suppliers. In other words, the application pattern of firm-generated content (FGC) has yet to be discovered in the research topics, which can be considered a gap in tourism content marketing research [62].

### 4.3 Co-keyword analysis

To explicitly examine thematic developments, the tourism content marketing research themes were divided into two phases: Phase 1, from 2007 to 2014, and Phase 2, from 2015 to 2022, based on when the number of articles spiked. The study selected a cut-off point of five for both stages, meaning that keywords that appeared together in each period at least five times appeared in the co-keyword network.

**Fig. 4:**
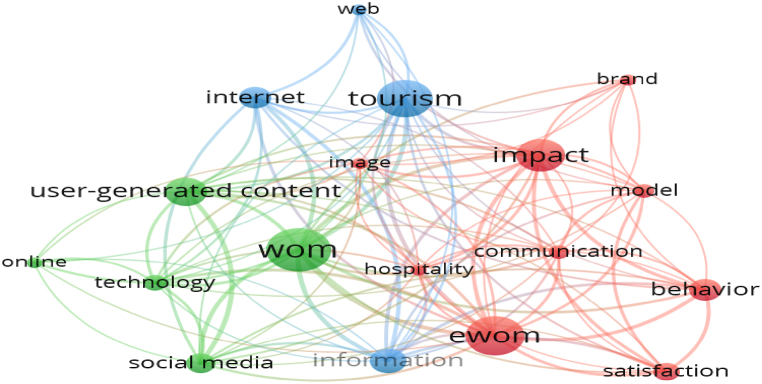
Visualized co-keyword network in tourism content marketing, 2007–2014.

From 2007 to 2014, 64 articles and 373 keywords were included in the co-keyword analysis. The results from VOSviewer software are presented in Fig. 4; 18 keywords meet the cut-off point criteria. At this stage, the co-keyword network is still sparse, demonstrating that the tourism content marketing theme in this period has not received much attention from a scholarly perspective. A total of three themes were created in the co-keyword analysis in Phase 1; an analysis of the representative keywords in each theme facilitated the naming of each theme presented in Table 4.

**Fig. 5:**
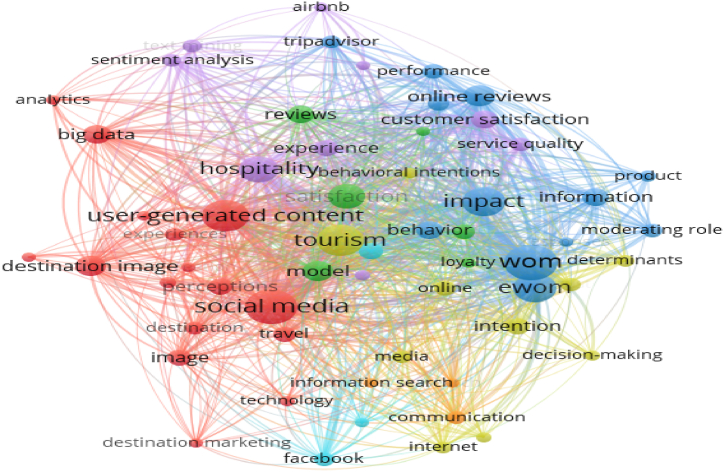
Co-keyword network in tourism content marketing in 2015–2022.

Phase 2 has more keywords than Phase 1 (Fig. 5). This indicates that, since 2015, tourism content marketing has received considerable attention from scholars. With 596 articles and 2821 keywords included in the co-keyword analysis for 2015–2022, a total of 241 keywords that met the cut-off point criteria were created and categorized under seven themes. These themes were analyzed and named, as shown in Table 4.

#### 4.3.1 Thematic evolution of studies in the field of tourism content marketing

This study systematically evaluated changes in keywords for each theme in both phases (Table 4). For Theme 1, “Implementation of online reviews,” in business performance, the concepts related to online reviews were developed based on WOM and eWOM models. The effects of reviews were specifically investigated based on motivations. The influence of Phase 2 extends to business aspects (e.g., “hotel performance,” “sales,” “price,” “ratings”) rather than an impact only on customer aspects, as in Phase 1. In Phase 2, the tendency to establish theoretical models to investigate the effects of reviews has become common. This suggests that a quantitative approach to this theme is gradually becoming more attractive than in Phase 1.

**Table 4:** Co-word clusters and representative words in two periods.

Themes	Keywords (Frequency)	
	2007–2014	2015–2022
Theme 1: Implementation of eWOM in hospitality	eWOM (18), impact (15), behavior (10), satisfaction (8), communication (6), brand (5), image (5), hospitality (5)	WOM (207), eWOM (138), impact (141), online reviews (79), behavior (58), performance (38), moderating role (33), hotel reviews (11), motivation (15), hotel performance (15), sales (14), hotels (13), price (12), ratings (11), reputation (9), booking (6), financial performance (5), management response (5), signaling theory (5)
Theme 2: User-generated content implementation	WOM (20), user-generated content (13), social media (9), technology (7), online (5)	social media (196), user-generated content (164), destination image (69), big data (61), perceptions (48), experiences (31), engagement (23), motivations (15), attitude (14), authenticity (14), personality (12), netnography (12), social network analysis (8), deep learning (6), photographs (6), travel blogs (5), geotagged photos (5), visual content analysis (5)
Theme 3: Communication and information search	tourism (17), information (11)	communication (30), information search (17), purchase intention (13), destination trust (6), travel intention (6), social networks (6), gender (5)
Theme 4: Customer behavior prediction model	N/A	satisfaction (103), model (69), reviews (57), antecedents (25), loyalty (21), intentions (18), attitudes (17), consumer-behavior (13), emotions (12), advertisement (11), choice (10), recommender systems (6), preferences (5), perception (7), credibility (12), attraction (6), brand personality (5), dissatisfaction (5), Chinese tourists (5)
Theme 5: Decision making process	N/A	tourism (166), intention (46), trust (32), decision-making (19), source credibility (13), planned behavior (12), online travel reviews (10), technology acceptance model (9), trustworthiness (9), consumer-generated media (7), perceived risk (8), customer loyalty (8), adoption (7), information quality (7), participation (6), PLS-SEM (6)
Theme 6: The issues related to user experience	N/A	hospitality (119), customer satisfaction (54), experience (45), service quality (34), sentiment analysis (31), text mining (30), involvement (19), sharing economy (18), perceived value (15), big data analytics (13), value co-creation (13), social media analytics (9), machine learning (8), customer experience (7), expectations (5), place attachment (5), business intelligence (5), latent dirichlet allocation (5)
Theme 7: The issues related to quality and management	N/A	quality (44), management (37), Facebook (34), customer engagement (17), knowledge (13), sustainability (11), communities (8), creation (7), revisit intention (6), decision (6), brand loyalty (5), mediating role (5),

*Note:* Numbers in parentheses are the frequency counts of keywords.

Based on Table 4, keywords representing financial impacts, theoretical modeling, and methodologies appear only in Phase 2. This reveals that developing a theoretical model to account for the effects of online customer reviews on a tourist destination's “financial performance” and how businesses exploit or deal with these effects is a trend that may continue.

In Theme 2 “UGC implementation,” the impact of UGC is discussed in both phases (Table 4). However, in Phase 2, the concept of UGC has expanded to a more visual form (image). The authors observed an increasingly in-depth investigation of UGC in Phase 2, with the appearance of many new keywords such as “perceptions,” “motivations,” “personality” (of users), and “authenticity” (of UGC). Based on the analysis of the co-keywords network, the authors suggest that future research should investigate the effects of visual content shared by users rather than text-based content such as travel stories from user-generated videos (UGVs) or travel experience images. In addition, focusing on analytical techniques specific to these visual UGC forms should also be considered. Aspects related to the characteristics and perception of these media forms can lead to breakthroughs in developing scientific flow in tourism content marketing.

In Theme 3, “Communication and information search,” information-related factors have developed into information-searching and communication in Phase 2 (Table 4). In Phase 2, the effects of communication and information-seeking activities on “purchase intention,” “destination trust,” and “travel intention” have been explicitly investigated. However, characteristics related to such activities have not been expressly confirmed. Identifying functional and affective characteristics formed from communication activities and information-searching online may be a potential research direction for the future.

Theme 4 is called the “Customer behavior prediction model.” The keywords in this theme only appear in Phase 2, which indicates that the effects of “reviews,” “advertisement,” “recommender systems,” and “preferences” on “satisfaction,” “loyalty,” “intentions,” “attitudes,” “consumer behavior,” “tourist satisfaction,” “destination choice,” and “dissatisfaction” are still in the development phase. Studies under this theme tend to focus on the “credibility” of the source, visitors' “perception,” “attraction,” and the formation process of tourists’ “emotion.” Most survey subjects are “Chinese tourists.” Constructing cognitive factors of emotional formation to predict visitor behavior through reviews can continue in the future. The awareness-interest-desire-action (AIDA) model and appraisal and cognitive emotion theory are potential theoretical foundations that can support this research direction.

Theme 5 is named the “Decision-making process.” Keywords in this theme indicate that travelers often use “online travel reviews” or “consumer-generated media” to make decisions. Research studies often adopt the approach of identifying “trust,” “perceived risk,” and “information quality” from reviews or information sources generated by visitors. Foundational theories such as “source credibility” or the “TAM” are commonly used to explain the above effects. Based on current research, the authors suggest that the SCT conceptual model approach to investigating tourists’ decision-making process through online travel reviews can be carried out in the future. However, it should be noted that the model is a multidimensional concept [76]. Understanding the model from different perspectives may open up potential research directions.

Theme 6 is named “Issues related to the user experience.” Studies under this theme tend to explore the effects of factors such as “perceived value,” “expectations,” “involvement,” and “place attachment” from visitors’ shared experiences on “customer satisfaction” and “service quality.” They are often built using the theoretical model of a “sharing economy,” “value co-creation,” and the conceptual framework of “business intelligence.” In addition, from Table 4, most studies related to this theme only focus on the hospitality field. Another significant finding is the practice of various analysis techniques (“sentiment analysis,” “text mining,” “big data analytics”). From developments in the research, future studies can approach the research theme from the perspective of a travel service provider or a tourist destination supplier. Furthermore, scholars can establish an application model of positive experiences as a destination marketing tool or a proposed model to improve negative experiences.

The final theme is named “Issues related to quality and management.” As described in Table 4, issues related to “quality” and “management” are often explored by determining the effects of “knowledge,” “communities,” and “creation” on “customer engagement,” “sustainability,” “revisit intention,” “decision,” and “brand loyalty.” Few studies have focused on establishing a “mediating role.” Based on the scientific development of the research theme, future studies can continue to expand factors related to “knowledge” and “creation” to improve the problems of “quality” and “management” in various aspects (e.g., “service quality,” “information quality” and “information management and control”). Investigating the impact of “customer engagement” on visit intention creates sustainable values for businesses could be a research direction to pursue. Meanwhile, a quantitative approach to the research theme or exploring the “mediating role” to understand issues related to “quality” and “management” should be considered.

### 4.4 Relationship between co-citation and co-keyword

**Fig. 6:**
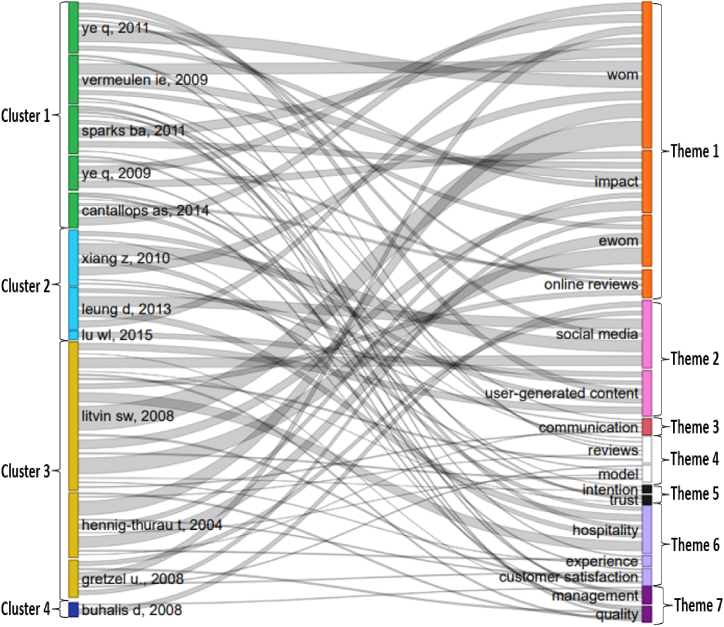
Relationship between co-citation and co-keyword. *Note:* The rectangle size represents the number of normalized articles, and the thickness of the links represents the number of co-citation and co-keyword ties.

The keywords in Fig. 6 represent the themes generated from the co-keyword analysis. Similarly, the authors (shown by the first author's name) are representative of the clusters generated from the co-citation network. According to Fig. 6, the impacts of “WOM,” “eWOM,” “online reviews,” “user-generated content,” “social media,” and issues related to “hospitality” are essential topics. These topics are derived from Vermeulen and Seegers [48], Ye et al. [49], Sparks and Browning [50], Leung et al. [54], Litvin et al. [58], Hennig-Thurau et al. [59] and Xiang et al. [68].

### 4.5 Keyword research trends in the period 2020–2022

**Fig. 7:**
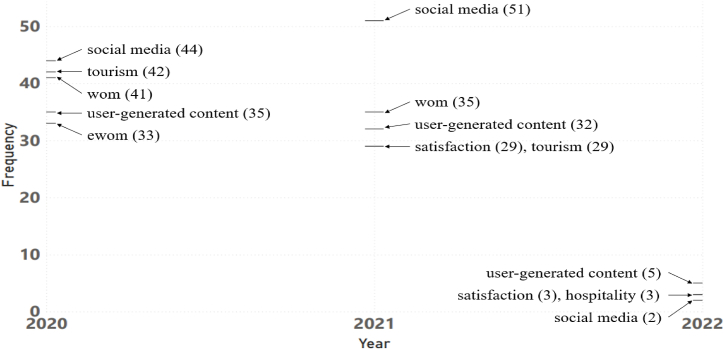
Most frequently appearing keywords in the period 2020–2021.

The most frequently appearing keywords in each year from 2020 to 2022 are presented in Fig. 7. The articles collected in 2022 are limited; thus, the keywords that appear are sparse. Two of the five keywords that appear the most frequently in 2020 and 2021 are repeated in 2022: “user-generated content” and “social media.” The former could arguably be the most prominent in 2022 (at least in the first quarter of 2022).

**Fig. 8:**
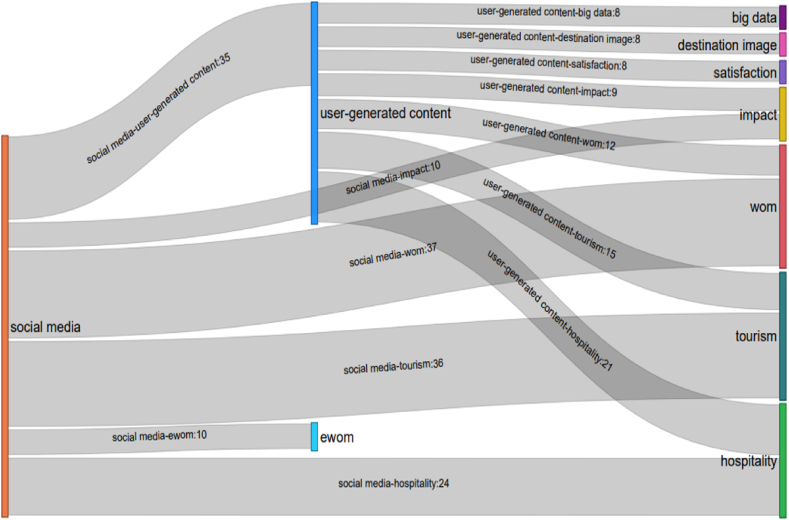
Relationship between “social media,” “user-generated content,” and other keywords in 2020–2021. Note: The rectangle size represents the number of occurrences for each keyword, while the thickness of the links represents the number of occurrences between keywords.

In addition, the two aforementioned keywords often appear together every year (2020–2022) (Fig. 8). The discovery of these keywords can be considered the most notable findings of the past three years. These two keywords represent the second theme in the co-keyword network analysis. The “UGC implementation” theme is likely the most promising trend to which scholars will pay attention in the future. The authors strongly believe that this theme still holds considerable research potential.

This study then synthesized potential research issues in publications related to UGC and “social” media. To generalize the values of the analytical results, this applies only to the publications with the most citations in 2020–2021 (Table 5).

**Table 5:** Synthesize potential future research trends from the co-keyword analysis.

No	Themes	Suggestions
1	Implementation of eWOM in hospitality	Develop studies investigating the effects of eWOM on financial performance and how businesses respond to eWOM activities.
2	User-generated content implementation	Expand investigation of impacts from other visual UGC platforms or types.
3	Communication and information search	Expand investigation of the functional and affective characteristics of tourism communication and information search.
4	Customer behavior prediction model	Research must focus on the emotional factor in building a model to predict visitor behavior.
5	Decision-making process	Exploit the different aspects of the SCT conceptual model.
6	The issues related to user experience	Categorizing and assessing the effects of positive and negative visitor experiences can be further exploited.
7	The issues related to quality and management	Elements related to knowledge and creativity should be extended to increase the company's understanding of management or quality-related issues. Besides, the sustainability factor should also be paid attention to because this factor is a trend in the tourism industry and all economic sectors.

## 5 Discussion and conclusion

Tourism content marketing is an important area that contributes to tourism development in the context of national economic development, especially when countries are generally trying to recover their economies, particularly tourism, following the COVID-19 pandemic. Understanding such marketing will help scholars and practitioners interpret and address the needs of the practice. This study identified the knowledge bases, research trends, gaps, and weaknesses in the research field to create a premise on which subsequent scholars can approach the field.

This study utilized bibliometrics. The authors compiled articles from 2007 to 2022 from the WoS database and a total of 659 qualifying publications were included in the analysis. Based on the number of publications each year, the results of the descriptive analysis revealed that the tourism content marketing sector is proliferating, especially after 2015. The articles were published in numerous journals worldwide. The most popular journal is *Tourism Management*, with 56 publications, followed by *Sustainability*, with 53 publications.

A co-citation analysis resulted in a co-citation network of four clusters. Cluster 1 considers the effects of eWOM and WOM on business performance. Cluster 2 examines the role of social media, UGC, and destination image formation. Cluster 3 covers the effects of eWOM and UGC on the decision-making process. Cluster 4 analyses opportunities and challenges. The results indicate that research on tourism content marketing has been shaped mainly by WOM and eWOM. According to Wang [77] and Zhao et al. [78], the research field has been built mainly on the literature on WOM and eWOM, combined with constructions borrowed from other models (e.g., TAM, TPB, and ELM). The findings from the co-citation analysis reinforce those of Leung et al. [79] and Ukpabi et al. [73]. Owing to the dominance of eWOM and WOM in the past, future studies may continue to apply them as theoretical underpinnings in tourism content marketing. Moreover, according to Yung et al. [80], the trend of using content marketing to provide different experiences of travel destinations and evoke positive emotions toward destinations is growing in importance and popularity. Thus, the use of emotional evaluation theories, for example, the theory of involvement [81], theories of memory [82,83], and appraisal theories [[84], [85], [86]], may be a potential approach to the research field that deserves considerable attention.

In addition, the authors did not find any clusters discussing the application model related to FGC, which could be explained by the fact that this topic is still in development. Therefore, it may be a shortcoming in the conceptual structure that requires special attention. The above discussions help answer the first research question: “What are the intellectual structures of tourism content marketing studies?”

A co-keyword analysis was conducted independently from 2007 to 2014 and from 2015 to 2022. The results in Table 4 demonstrate that the research in Phase 2 is becoming more diverse and specialized than in Phase 1. Based on thematic analysis, tourism content marketing research is reaching maturity, with an increasing number of new publications. According to Fotis et al. [87], tourists search for travel information, share travel experiences, and provide feedback based on online reviews. Tourists often perform these activities at every stage of the travel process, pre-, during-, and post-trip [87]. Accommodation enterprises can use online reviews to engage with customers and determine consumer opinions based on building an online presence [50]. Such online reviews influence booking intention and earnings [88]. Given the multidimensional usefulness of online reviews (travelers and businesses), their popularity in the academic context is not surprising [89]. However, travelers’ online reviews are not only the forms of comments and ratings on online platforms such as Facebook and hotel websites, Booking.com, TripAdvisor, and Agoda [ [58,62]], but also other complex forms such as blogs, vlogs, pictures, or travel narrative posts [90]. Such content is user-generated (UGC). As a result of the increasing demand for travel information search, the concepts related to tourism content have become diverse and rich [91]. Consequently, techniques for analyzing their effectiveness and impact also become complex, such as big data, netnography, social network analysis, deep learning, and visual content analysis, which were found under the theme “UGC implementation.”

The application of big data and deep learning is growing and strongly impacts tourism [92]. Techniques such as artificial neural networks, support vector machines, and deep learning are applied to natural language processing tasks [92]. Most of these techniques were developed more than 50 years ago, but the growth of big data and computer processing capabilities today has caused these techniques to be revived and widely used [93]. The authors expect these techniques to continue to be developed and applied in the text processing of UGC (e.g., online travel reviews), creating a premise for developing novel sub-techniques such as neural network deployment and multilayer neural network (deep learning) models. In addition, the emergence of netnography analysis is a development in applying innovative research techniques to the “UGC implementations” theme because it is applied to hear and understand the voice of personal digital informants that big data analysis cannot do well [94]. Moreover, although modern research techniques such as social network analysis and visual content analysis are commonly applied, the research themes require more innovation from advanced methods, as has happened with netnography.

The travel industry relies heavily on WOM activity among users [87]. Before the advent of the Internet, WOM activity often occurred between relatives [95]. With the arrival of the Internet, consumers turned to consult information from other online consumers [95]. Presently, the decision-making process, behavior, and online experiences related to tourist destinations are shaped by the content consumers read, which can be seen as a significant development in tourism [96]. In addition, the growth in the demand for tourist information and the diversity of available information sources significantly change the nature of the impact of information collected on potential visitors [97].

Furthermore, according to Ayeh et al. [71], each form of content has different cognitive characteristics and effects. Therefore, changes in demand and the variety of content formats have likely resulted in different visitor activities after the information-search stage. This explains the explosion of themes that appeared after 2014. Scholars approach the research field using empirical studies to investigate visitor behavior and decision-making through online information sources or travelers’ shared online experiences. The appearance of new keywords related to the characteristics and quality of information sources and emotional factors shows an increasing need for tourists to search for tourist information. Their requirements have become more rigorous and thorough. This change in demand entails differences in the techniques and methods of analyzing the effectiveness of tourism-information sources, as shown by new keywords such as text mining, sentiment analysis, big data analytics, machine learning, and latent Dirichlet allocation. These findings suggest that the studies in theme 6 apply various analytical techniques borrowed from data science. According to Li et al. [98], current analytic techniques face challenges related to the unstructured and complex features of shared online experiences. Although analytic techniques have been discovered to be valuable, new techniques are needed to understand the meaning of hidden topics in unstructured data for research context. Several studies attempt to innovate analytical techniques by integrating the listed techniques above [ [99,100]]. However, the authors expect that the availability of new big data sources will further spur innovation and creativity in tactics for collecting user experience data. San and Lai [101] suggest that natural language processing, location-based data, and text analysis would be the new trend. Such methods can be applied with data sources that accommodate other forms of user insights [102]. In addition, Kim and So [37] argue that the fundamental nature of user experience can be reflected through several methodologies, such as electroencephalography, electrodermal activity, and the experience sampling method.

In addition, Theme 7 reveals that quality and management are the subjects discussed in online content. This theme reflects that visitors are interested in the characteristics of the information source and the value derived from the information. Tourists require information about what they can expect to experience at the destination, such as the quality of service [103] or the quality of the destination [104].

From the perspective of organizations, online content can be used to improve operations or management strategies. Muritala et al. [39] suggested that online content was used to investigate visitor responses to hotel sustainability measures and their impact on the guest experience. However, the results of the current study extend the above findings. Tourism businesses can draw profound experiences from shared tourist information sources to improve quality and create a premise to satisfy visitors' needs, thereby creating sustainable development.

Once again, the authors emphasize that tourism content marketing is in a period of solid growth. Therefore, the research field will require innovative activities in the future. Using more advanced methodologies, such as big data analytics, is a starting point. For example, analyzing online content from a single data source has become obsolete. Therefore, to enhance generalization and contributions to the research field, scholars should synthesize and analyze data from multiple platforms [105]. In addition, empirical research should apply new theoretical models such as dual system theory [106] or conceptual models of tourism inspiration [107].

In addition, an evaluation of the development of the research themes allows the authors to propose potential future research directions, summarised in Table 5. The co-keyword analysis answers the second research question, “What are the major research themes in tourism content marketing?”.

Identifying the relationship between co-citation and co-keyword analysis allowed the authors to pinpoint pioneering studies that have led to and shaped current research trends. Specific results reveal that Themes 1, 2, and 6 often used scientific knowledge from studies by Vermeulen and Seegers [48], Ye et al. [49], Sparks and Browning [50], Leung et al. [54], Litvin et al. [58], Hennig-Thuau et al. [59] and Xiang et al. [68] (see Fig. 6). These results help answer the following research question: “What are prominent scientific insights most cited to shape each research theme?”

Analysis of the most popular keywords for 2020–2022 determines that the theme “UGC implementation” is the most prevalent research trend to develop in the future. Specific research issues identified for the future are summarised in Table 6. These results allow the authors to address the third research question: “What is the most potential theme that could be developed in the future?”.

**Table 6:** Summary of suggestions related to “social media” and “user-generated content.”

No.	Articles	Suggestions
1	Pop et al. [109]	• Explore and measure the factors affecting the travel planning behavior of Generation Y. • Consider applying other factors that may influence tourism decision-making, for example, control of perceived behavior, demographic factors, perceived service quality, and loyalty. • Investigate tourists' trust in social media influencers to determine trust in tourism businesses or brands.
2	Atsız et al. [110]	• Investigate host motivations and perceptions towards the food experience aspect. This approach should be taken by a quantitative method. • Consider entrepreneurship from a conceptual model of the meal-sharing economy, which could be a curious topic to be determined by future studies. • Explore the role of foodservice quality and its impact on visitor intention/behavior in the context of food experience. • Explore the antecedent role of ‘belief.'
3	Dedeoğlu et al. [111]	• Investigate the factors that stimulate visitor adoption of UGCs or the importance of UGCs on their behavior. • Compare the effects of UGC shared on social networking sites with third-party web platforms (TripAdvisor) where interpersonal interactions are lacking.
4	Liang et al. [112]	• Investigate possible effects of MGC (marketer-generated content) on the increase in UGC volume. • Classify positive and negative UGCs using linguistic methods such as content or sentiment analysis, then explore the effects of the MGC on these positive and negative UGCs.
5	da Mota & Pickering [113]	• Investigate the characteristics of the user or the content and the motivation behind the posting behavior. • Expand case study regions/countries such as Brazil, China, and Russia, where attractive tourist destinations but the scope of the study is generally sparse. • Expand social media platforms for investigation, such as Twitter and Flickr. • Exploit other content analysis techniques such as image content analysis or big data.
6	Anagnostopoulou et al. [114]	• Identify the control factors for the qualitative characteristics of visitors. For example, impressions made on first-time vs. repeat travelers, business vs. leisure tourists, or local vs. international guests. • Classification of effects from negative or positive reviews should be done in the future to avoid problems associated with dual valence reviews.
8	Van der Zee et al. [115]	• Expand investigation of impacts from other types of visual UGC.
9	Jiménez-Barreto et al. [116]	• The online destination brand experience and brand authenticity should also investigate other stages of the tourist experience (during and after the visit). The approach should be taken using both qualitative and quantitative methods. • Investigating cultural differences in the approach to the brand platform to determine its impact on authenticity is recommended in the future.
11	Pourfakhimi et al. [117]	• Expanding investigation into the impact mechanisms of intelligent technologies such as artificial intelligence, virtual reality, and augmented reality in tourism content marketing.
12	Lam et al. [118]	• Expand the investigation into the impact of other UGC platforms, such as blogs and YouTube, during and after the trip on destination image formation. • Investigate the effects of FGC or company-owned content on tourist destination image formation should be focused on future efforts.
13	Marine-Roig & Huertas [119]	• Add the “lack of safety” element to the investigation of tourist destination images.

The authors identify knowledge gaps in tourism content marketing based on scientific mapping and analytical results. First, identifying an effective communication tool or strategy will improve business performance and save resources. However, until now, the form of content marketing (text, image, video, and others) or source of information that is most effective in the field of study has not been determined. Second, the effectiveness of content marketing not only generates revenue for businesses and brings competitive advantages for their sustainable development. However, the research field has not yet thoroughly explored the competitive aspect. Third, although content marketing brings many economic and other benefits to businesses, it can lead to communication crises. Meanwhile, although content-marketing activities are being conducted in the tourism industry, the problems related to media crises have not been identified. Due to these scientific gaps, future scholars are encouraged to approach the research area based on the suggestions in Table 5, Table 6

These findings will be of interest to tourism content marketing scholars as they can understand the current state of knowledge in the context of the tremendous growth in the volume of research. In addition, the results from the thematic analysis will allow scholars to identify potential trends and prioritize efforts for the hottest and most prominent trends, such as “UGC implementation.” Furthermore, using the Power BI tool to visualize the “pioneers” of each research trend will assist scholars in identifying credible sources for their literature review that can enhance the current understanding of the trend.

The findings also give professionals and marketers insights into the concepts and issues they confront daily. On the one hand, the study presents an overview of tourism content marketing trends, which helps managers determine the demands for information search and requirements in content characteristics to enhance effective marketing. On the other hand, the trends mentioned allow marketers to gain knowledge related to the current trend in visitors' virtual-travel experiences. Specifically, enterprises should develop marketing strategies aligned with tourists' increasingly demanding and diverse needs for online experiences. In addition, the prolonged COVID-19 pandemic allows travelers to create a habit of virtual-travel experiences through firms’ content-marketing platforms (platforms owned by or in collaboration with firms) [108]. This phenomenon can lead to last-minute bookings [44]. According to Toubes et al. [44], when the pandemic is about to end, businesses can launch promotional activities to attract visitors. Therefore, enterprises should reserve sales and booking policies to deal with this problem when the pandemic ends. Finally, the results from this study provide organizations with an opportunity to apply content-marketing analytics methods and techniques such as machine learning, deep learning, sentiment analysis, text mining, and big data analytics. Businesses can use them to support decision-making for improvement and future business-strategy planning.

### 5.1 Limitations and future works

Despite significant efforts to complete this study, limitations related to data collection and analytical shortcomings cannot be avoided. First, the data on WoS did not include book records. Therefore, the research results do not comprehensively outline the research area and may be biased. The inclusion of book records may change the co-citation and co-keyword networks. Such a shift could lead to new meaningful discoveries in the research field that scholars should endeavor to implement in the future. Second, due to the inaccessibility of the full text, a few studies were excluded from the formal analysis. These studies could very well contain meaningful data on tourism content marketing. Regarding this problem, the authors were constrained by the functionality of the WoS database system. Thus, the study could not address this challenge. Third, the use of only one database may result in findings that are not representative of the entire field. Future studies may consider using a variety of databases (e.g., Scopus) and analyzing all the records collected.

Finally, according to Chamboko-Mpotaringa and Tichaawa [6], different countries have different levels of digitization. In this regard, the technological innovation capabilities of tourism businesses in developed countries are more advanced than in developing countries [120]. This distinction requires marketers to absorb and learn from the experiences of developed countries. Therefore, scholars and marketers should conduct bibliometric studies on the national scale for developing tourism destinations (e.g., Vietnam and Thailand). It would help understand the scientific knowledge and current content-marketing trends and recommend appropriate marketing strategies to stay competitive.

## Author contribution statement

Phuong Minh Binh Nguyen: Conceived and designed the experiments; Performed the experiments; Analyzed and interpreted the data; Wrote the paper.

Xuan Lan Pham, Associate Professor: Conceived and designed the experiments; Contributed reagents, materials, analysis tools or data; Wrote the paper.

Giang Nu To Truong, Ph.D: Contributed reagents, materials, analysis tools or data; Wrote the paper.

## Funding statement

This research is funded by University of Economics Ho Chi Minh City, Vietnam.

## Data availability statement

Data will be made available on request.

## Declaration of interest’s statement

The authors declare no competing interests.
